# Uptake of Fungicide Fluopyram from Soil by Scallions during Greenhouse Cultivation

**DOI:** 10.3390/foods12101996

**Published:** 2023-05-15

**Authors:** Myung-Sub Yun, Hoon Choi

**Affiliations:** 1 Department of Life and Environmental Sciences, College of Agriculture and Food Sciences, Wonkwang University, Iksan 54538, Republic of Korea; 2 Hanearl Science Ltd., Sungnam 13207, Republic of Korea

**Keywords:** fluopyram, bioconcentration factor, scallion, rotational crop, uptake

## Abstract

Unintentional pesticide contamination in rotational crops, often caused by soil contamination from pesticide use in the preceding crops, is a major concern in a positive list system. The residue and dissipation pattern of fluopyram in soil and scallions were investigated to evaluate the uptake of fluopyram from the soil by scallions. In addition, the management concentration in soil (MC_soil_) was calculated based on bioconcentration factors (BCFs) and the maximum residue limit (0.2 mg/kg) in leaf-and-stem vegetables. In a field experiment, plots in two different trials, A and B, were treated with 0.06 g fluopyram/m^2^ and maintained for 30 days according to OECD guidelines. Scallion seedlings were cultivated for 48 days. Soil samples were taken at three different time points: DAP (Days after planting) 0, 34, and 48. Scallion samples were collected at five different time points: DAP 20, 27, 34, 41, and 48. The initial amounts of fluopyram in soil at DAP 0 were 0.94 ± 0.03 and 0.96 ± 0.04 mg/kg in trials A and B, respectively. The half-life of fluopyram in the soil was 87–231 days. Fluopyram uptake by the roots increased over time, but fluopyram residue in the scallions decreased due to the dilution effect caused by an increase in plant weight. The residues in the scallions at DAP 48 were 0.22 ± 0.01 and 0.15 ± 0.01 mg/kg in trials A and B, respectively. The BCFs of scallions for fluopyram were 0.21–0.24 (trial A) and 0.14–0.18 (trial B). The MC_soil_ was proposed as 0.8 mg/kg, and may be utilized as a safe management guideline for precautionary practices to cultivate safe rotational crops.

## 1. Introduction

Pesticides sprayed on crops attach to their surfaces, fall to the soil, and either remain in the soil or transported to water systems [1,2]. Pesticides decompose and dissipate over time through various physico-chemical and biological processes, but in some cases a significant amount of the pesticide can be introduced into the soil, resulting in soil contamination. When plants are grown in contaminated soil, trace amounts of pesticides can be taken up by the plant roots and transferred to other parts of the plant [3,4], leading to pesticide contamination of the plants. Leafy vegetables and leaf-and-stem vegetables are more prone to this type of contamination compared to fruits, as they have a higher chance of accumulating pesticides due to their edible parts [5,6,7]. This accumulation can result in unintentional contamination of agricultural products and pose health risks to consumers.

Fluopyram is a benzamide-based fungicide that inhibits the function of succinate dehydrogenase (SDH) inside the mitochondrial membrane, thereby controlling pathogenic fungi in plants [8]. Fluopyram is widely used in Korea to control fungal growth, such as grey mold (*Botrytis*) and powdery mildew in fruits and vegetables. The organic carbon adsorption coefficient for fluopyram (K_oc_), which is a representative indicator of the mobility of pesticides in soil, is 233–400 mL/g, indicating a moderate level of mobility [9]. Additionally, fluopyram is heat-stable and very stable during hydrolysis and photolysis in acidic, neutral, and alkaline conditions, and the half-life in soil was reported as 119 days [10]. Therefore, owing to high soil persistence and moderate mobility of fluopyram, there is a strong possibility that it would remain in the soil and be absorbed into crops. Furthermore, the Korean cropping system has the characteristics of a short uncultivated period and frequent continuous/mixed cultivation, which has increased the possibility of pesticide uptake and contamination of rotational crop plants. In a survey on pesticide residues conducted by the Korean Ministry of Agriculture, Food and Rural Affairs in 2019, the residual amount of fluopyram was the highest among 13 unregistered pesticides detected in scallions, a leaf-and-stem vegetable [11]. A positive list system (PLS) has been implemented to strengthen domestic pesticide safety management. In this system, the maximum residue limit (MRL) applies a standard legal limit of 0.01 mg/kg to all non-registered pesticides. As a result, unintentional contamination of agricultural products due to pesticides remaining in the soil has become a more considerable problem than before the implementation of PLS. Therefore, it is necessary to establish safety management standards for residual pesticides in the soil by evaluating the uptake and translocation of soil residual pesticides into crops.

Scallion (*Allium fistulosum*) belongs to the genus *Allium* of the Liliaceae family and is a major leaf-and-stem vegetable in Korea with a cultivation area of 17,170 ha and an annual production of 463,721 tons [12]. In addition, unintentional persistence of fluopyram has been reported for scallions, raising safety concerns [11]. To the best of my knowledge, there are no previous reports on the potential for fluopyram residues to be present in scallions as a result of uptake from soil. Therefore, this study assesses the uptake of the fungicide fluopyram from the soil by scallions and proposed a management concentration for fluopyram in the soil to ensure the safety of agricultural products. The residual patterns of fluopyram in soil and scallion were analyzed, and the bioconcentration factor (BCF) was calculated as the absorption rate of fluopyram into scallions.

## 2. Materials and Methods

### 2.1. Reagents and Materials

Fluopyram (purity: 98%) was purchased from Sigma-Aldrich (St. Louis, MO, USA). The structure and physico-chemical properties of fluopyram are shown in Table 1 [10].

As a crop protection product, 40% suspension concentrate (SC) of fluopyram (Mercury, Bayer Crop Science Co., Ltd., Daejeon, Republic of Korea) was selected and purchased from a commercial agrochemical vendor. The analytical solvents, including acetonitrile and methanol, were purchased from J.T. Baker (Center Valley, PA, USA). High-performance liquid chromatography (HPLC) grade formic acid (purity: 99%) was purchased from Wako (Osaka, Japan) and ammonium formate (purity: ≥97%) was obtained from DAEJUNG (Siheung, Republic of Korea). Reagents used in the quick, easy, cheap, effective, rugged, and safe (QuEChERS) pretreatment method were purchased from Agilent Technology (Santa Clara, CA, USA). QuEChERS extract pouches (EN 15,662 method extraction kit; 4 g MgSO_4_, 1 g NaCl, 1 g sodium citrate dehydrate, and 0.5 g sodium hydrogencitrate sesquihydrate) were used for partitioning, and dispersive solid phase extraction (d-SPE, 2 mL, 150 mg MgSO_4_ and 25 mg primary secondary amine (PSA)) was used for purification. A 100 mg/L stock solution was prepared by dissolving 10.20 mg of standard fluopyram (purity: 98%) in 100 mL of acetonitrile, and serially diluted using blank sample extracts to construct the matrix-matched working solutions at 1, 2, 5, 10, 20, 50, and 100 μg/L, which were used to correct for any matrix effects during the instrumental analysis. The stock and working solutions were stored at −20 °C in a freezer.

### 2.2. Field Experiment

The field experiment was carried out at a cultivation facility located in Jeonju, Jeollabuk-do, Republic of Korea (35°52′09″ N 127°06′30″ E). For a test crop, a Heukgang cultivar (*Allium fistulosum*, Farm Hannong Co., Ltd., Seoul, Republic of Korea) was cultivated in a greenhouse and scallion seedlings were used for field experiments 63 days after germination. For the field experiments, two different trials, A and B, were used, each with an area of 30 m^2^ (2 m × 15 m), and each equally divided into three repeating plots. Each trial was separated with a 6 m^2^ (2 m × 3 m) buffer zone. The physico-chemical properties of the soil were analyzed according to the official soil analysis methods outlined by the rural development administration (RDA) [13] and are indicated in Table 2.

The spray suspension was prepared by mixing 5 mL of 40% Mercury SC (suspenstion concentrate) with 20 L of water and sprayed on the trials using a power sprayer (MSB1500Li, Maruyama, Japan). The application rate of fluopyram was 0.06 g a.i. (active ingredient)/m^2^, which was the maximum seasonal rate determined from the maximum label rate and the maximum number of applications according to OECD (organization for economic co-operation and development) guidelines [14]. After application, the soil was homogenized to a depth of 10 cm. The plots were irrigated by spraying 100 L of water on each trial once a week to maintain 20% soil moisture content and maintained for 30 days (PBI-30; Plant back intervals) in a greenhouse. The seedlings were planted at a density of 15 × 15 cm after tillage to a depth of 15 cm and cultivated for 48 days. In each trial, 100 L of water was drip-irrigated every two days during cultivation. During the cultivation period, CAS data loggers (EL-21CFR-2-LCD, Lascar electronics, PA, USA) were used to monitor temperature (16.2–31.0 °C) and humidity (48.3–86.7%) in the facility.

### 2.3. Sample Collection and Preparation

At least 1 kg of topsoil (a depth 0–10 cm) was collected on the day after planting (DAP 0—days after planting), 34 days after planting (DAP 34), and 48 days after planting (DAP 48) using a soil sampling auger (diameter, 2.3 cm; and height, 10 cm). The soil samples were sieved using a <2 mm diameter sieve, and then stored at −20 °C until analysis. The moisture content of soil samples was determined by calculating the difference in soil weight before and after drying at 105 °C for 24 h. Scallion samples were collected on the 20, 27, 34, 41, and 48 days (100% mature) after planting (DAP 20, 27, 34, 41, and 48). The individual weight and height of each sample were measured to compare the growth status at the time of sample collection. After removing the root of the scallion and adding dry ice, samples were homogenized using a blender (Blixer^®^ 2, Lobot Coupe, Montceau-les-Mines, France), and then stored at −20 °C until analysis.

### 2.4. Extraction and Purification of Fluopyram

The residual analysis of fluopyram in soil and scallion samples was carried out based on the QuEChERS EN method for rapid analysis. Soil (5 g) or scallion samples were weighted and placed into 50 mL conical tubes. Thereafter, 20 mL of acetonitrile was added for extraction and samples were shaken at 1300 rpm for 3 min using a high-speed shaker (2010/Grinder, SPEX^®^ Sample Prep, Metuchen, NJ, USA). Subsequently, QuEChERS extract pouches (4 g of MgSO_4_, 1 g of NaCl, 1 g of sodium citrate dehydrate, and 0.5 g of sodium hydrogencitrate sesquihydrate) were added, followed by shaking at 1300 rpm for 90 s. After centrifugation at 4000 rpm for 5 min (Combo-408, Hanil Science Inc., Gimpo, Republic of Korea), a 1 mL aliquot of the supernatant was transferred into a d-SPE tube containing 150 mg of MgSO_4_ and 25 mg of PSA for purification. After shaking at 1300 rpm for 90 s and centrifugation at 13,000 rpm for 3 min, the supernatant was filtered through a 0.2 μm syringe filter. Thereafter, the filtrate (800 μL) was mixed with acetonitrile (200 μL). A 5 μL aliquot of the mixture was injected into a high-performance liquid chromatography-tandem mass spectrometer (HPLC-MS/MS).

### 2.5. Instrumental Conditions for HPLC-MS/MS Analysis

A Shimadzu Nexera HPLC coupled to a Shimadzu LCMS-8045 triple quadrupole mass spectrometer (Shimadzu Corp., Kyoto, Japan) was used for the analysis of fluopyram in soil and scallion samples. The chromatographic separation was achieved using a Cadenza C18 column (150 mm × 2.0 mm, 3 µm, Imtakt, Portland, OR, USA) at 40 °C with a 0.3 mL/min flow rate. The mobile phase was composed of water (Solvent A) and methanol (Solvent B), containing 0.1% formic acid and 10 mM ammonium formate, respectively. The gradient system of the mobile phase was programmed at a 0.3 mL/min flow rate as follows: 0–2 min, 20% B; 2–5 min, 20–80% B; 5–10 min, 80% B; 10–13 min, 80–20% B; 13–17 min, 20% B. The injection volume was 5 µL. MS/MS detection was achieved in positive ion mode with electrospray ionization (ESI). The source parameters were optimized as follows: desolvation line temperature, 250 °C; interface temperature, 300 °C; heat block temperature, 400 °C; nebulizing gas, 3 L/min; heating gas (air), 10 L/min; drying gas (N_2_), 10 L/min. In positive ion mode, *m*/*z* 396.8 was detected as the precursor ion, and *m*/*z* 173.0 and 144.9 were selected as the quantifier and qualifier at 30 and 50 V of collision energies, respectively. Instrument control, data acquisition, and processing were performed by the Shimadzu LabSolutions software package (ver. 5.97, Shimadzu Corp., Kyoto, Japan).

### 2.6. Method Validation for Quality Assurance

The analytical method was validated using the following parameters: matrix-dependent limits of quantitation (MLOQs), linearity of matrix-matched calibration curve, and recovery efficiencies, as recommended by the European SANTE/12682/2019 guideline [15]. The instrumental limit of quantitation (ILOQ) was the concentration level with signal-to-noise (S/N) ratio of 10. ILOQ of fluopyram was computed by analyzing matrix-matched working solutions at 0.01–10 μg/L. The MLOQs were determined from the ILOQ, injection volume, dilution factor, sample amount, and extract solvent volume [16]. The linearity was evaluated using the linear regression and coefficient of determination (R^2^) at 1–100 μg/L of the matrix-matched working solutions. A calibration curve using matrix-matched working solutions was utilized to quantify the amount of fluopyram in the soil or scallion samples using an external standard calibration method. Procedural blanks and spiked blanks were included as quality control (QC) samples in each batch instrumental analysis. Recovery efficiency assays were performed by spiking standard solutions into control samples at 0.01 (MLOQ), 0.1 (10 MLOQ), and 1.0 (100 MLOQ, only soil) mg/kg, in triplicate. The mean and relative standard deviation (RSD) of the recovery efficiency were calculated to evaluate the accuracy and precision of the analytical method.

### 2.7. Dissipation Constant and Half-Life for Fluopyram in Soil and Scallions

The dissipation constants for fluopyram in soil and scallion were calculated using a first-order kinetic model using Equation (1) [7,17,18]:

*C_t_* = *C*_0_ × e^−*λ*t^, (1)

where *C_t_* is the concentration of fluopyram in soil or scallions at time *t* (day), *C*_0_ is the initial concentration of fluopyram in soil or scallion at DAP 0, and *λ* is the dissipation constant. The half-life (DT_50_) of fluopyram was then calculated using Equation (2) [7,18]:

DT_50_ = ln (2)/*λ* = 0.693/*λ*, (2)

The time-dependent residues of fluopyram in soil and scallions were evaluated using the F-test, regression equation, and regression coefficient (R^2^). The mean and 95% confidence interval (95% CI) of the dissipation constant (*λ*) were calculated using regression analysis. Statistical analyses were performed using the statistical program, SPSS 18.0 (SPSS Inc., Arming, NY, USA).

### 2.8. Calculation of BCFs and Management Concentration in Soil

The concept of BCF was used to calculate the uptake rate of residual fluopyram from the soil into crops [17,18,19]. The BCF refers to the ratio of the concentration in the organism to the concentration in the environment, in the process of accumulating a specific pollutant in the organism. The BCF was calculated using Equation (3):

BCF = C_plant_/C_soil_, (3)

where C_plant_ is the residual concentration in scallion at DAP 48, which indicated a full-grown (100% mature) scallion, and C_soil_ is the residual concentration in soil on the day of planting (DAP 0).

The management concentration in soil (MC_soil_), which represents the concentration of fluopyram in the soil where the residual pesticide in the crop at harvest does not exceed the MRL, was calculated using Equation (4):

MC_soil_ = MRL_plant_/BCF, (4)

where the MRL_plant_ for fluopyram in leaf-and-stem vegetable by unintentional contamination is 0.2 mg/kg [20].

## 3. Results and Discussion

### 3.1. Growth of Scallions during the Experiment

**Figure 1:**
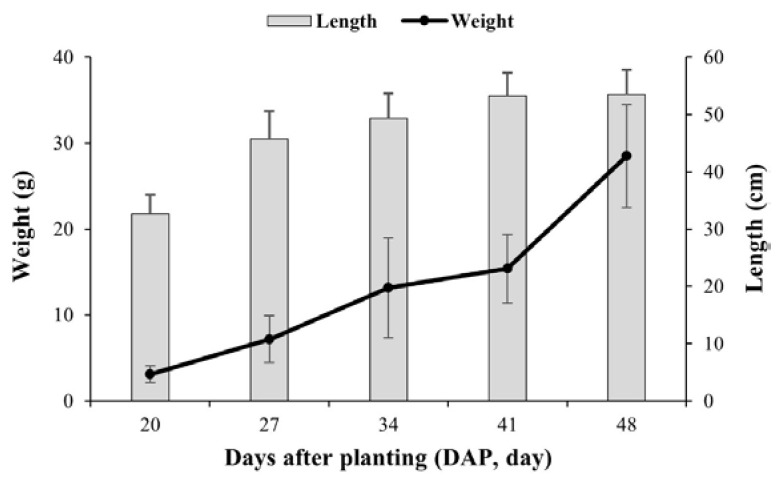
Growth characteristics of scallions during the cultivation period.

The growth characteristics of scallions are shown in Figure 1. Scallion length increased up to DAP 34, but showed no significant increase after DAP 41. Conversely, scallion weight steadily increased during the cultivation period to approximately 9-fold by the DAP 48, as compared to DAP 20. These results highlighted the general growth characteristic of the scallion: as harvest time approaches, the length of the scallion does not increase, but the plant becomes thicker and heavier.

### 3.2. Validation of the Analytical Method

The method validation was performed according to the European SANTE/12682/2019 guidelines [15]. During the analysis, no peak was detected at the retention time of fluopyram, indicating that there were no interferences affecting its detection. An analyte signal during ionization is enhanced or suppressed due to matrix components co-eluted with the analyte of interest, which is known as the matrix effect. Therefore, a matrix-matched calibration is necessary to compensate for the matrix effect during MS analysis. The MLOQ of fluopyram was calculated to be 0.01 mg/kg for soil and scallions.

The linearity of the matrix-matched calibration curves was good, with R^2^ > 0.9996 in soil and scallions, which satisfied the criteria of >0.98. As shown in Table 3, the average recovery efficiencies from soil and scallions were 71.3–96.3% with RSDs ranging from 0.4 to 2.2%, which was satisfactory with a recovery of 70–120% and reasonable RSDs ≤ 20%. These results demonstrated that the analytical method developed in this study had satisfactory precision and accuracy for quantifying fluopyram residues in soil and scallions.

### 3.3. Dissipation of Fluopyram in Soil

To investigate the uptake/translocation of pesticide from soil to rotational crops, it is necessary to understand the residual characteristics in the soil by examining pesticide application during the cultivation of the preceding crops. According to the OECD guidelines [14], a suspension solution of fluopyram should be prepared at the maximum label rate (dilution factor 4000) and applied to bare soil for the maximum number of applications (three times). However, the guidelines recommend a single application of the pesticide suspension at the maximum seasonal rate, rather than multiple applications for practical purposes. Therefore, in this study, fluopyram was applied to soil at a treatment level of 0.06 g a.i./m^2^. The OECD guidelines suggested plant-back intervals (PBIs) of 30 and 60 days. However, considering the domestic cropping system with a short uncultivated period, the PBI in this study was set at 30 days. The residual amount of fluopyram in soil was calculated in dry weight by correcting for moisture content. The moisture content of soil samples was in the range of 16.2–28.4%. The average residues at DAP 0 were 0.94 and 0.96 mg/kg in trials A and B, respectively, which decreased to 0.80 and 0.69 mg/kg at DAP 48, representing 84% and 72% of the initial residual amount, respectively. The DT_50_ values of fluopyram in soil were 231 (95% CI, 139–347) days and 87 (95% CI, 63–173) days in trials A and B, respectively. These were 2.4–14.6 times longer than 15.8–24.8 days (watermelon) and 36 days (tomato and bell pepper) in soil during fruit vegetable cultivation [7,21]. Generally, pesticides degrade and dissipate in soil owing to biological factors, such as plants and microorganisms, and non-biological factors, including rainfall, irrigation, hydrolysis, and photolysis [2,22]. However, our results showed that scallion cultivation gave no significant reduction in the fluopyram residue in the soil.

### 3.4. Uptake of Fluopyram by Scallions from Soil

**Figure 2:**
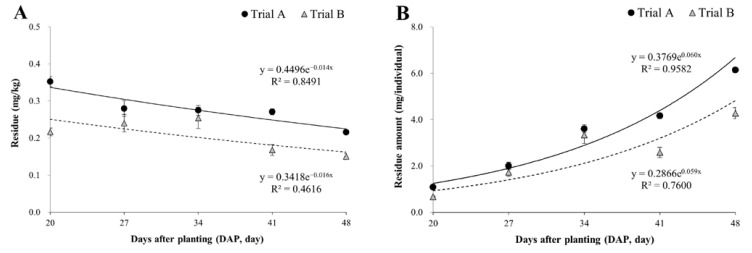
Time-dependent residual patterns of fluopyram in scallions. ((**A**) including dilution effect; and (**B**) excluding dilution effect).

The residual levels of fluopyram in scallions are shown in Table 4. Fluopyram residues at DAP 20 were 0.35 mg/kg and 0.22 mg/kg in trials A and B, respectively. As the plants reached maturity at DAP 48, residual levels decreased to 0.22 mg/kg and 0.15 mg/kg in trials A and B, which represented 61% and 69.5% of residues at DAP 20, respectively. The dissipation constants calculated using regression analysis were 0.014 (trial A) and 0.016 (trial B), respectively, and there was no significant difference between them (Figure 2A).

Pesticide residue in crops decreases due to reduction factors such as respiration, volatilization, metabolism, and weight increase through growth [23,24]. In contrast to previous research indicating an increase in residual amounts of fluopyram in tomato and pepper leaves during the cultivation period [7], this study found that decreased fluopyram residual levels in scallions were primarily due to the dilution effect resulting from the 9-fold increase in weight over the 24-day growth period (Figure 1). The residual amount (mg/individual) was calculated to determine the dissipation characteristics, while excluding the dilution effect. As shown in Figure 2B, there was a 5.6 to 6.4-fold increase in individual residues during the 24-day growth period. These results indicated that fluopyram uptake by the roots was greater than dissipation by metabolism, respiration, and other processes. The water solubility and the octanol/water partition coefficient (log K_ow_) of pesticides are important indicators of their mobility in soil and transportation in plants, since pesticides dissolve in the soil water, also known as the bioavailable portion of soil, and are taken up by plant roots and translocated to the aboveground tissues through xylem [4]. Fluopyram is classified as readily soluble, with a water solubility of 16 mg/kg and hydrophobic with log K_ow_ of 3.3 [10]. Considering its physico-chemical properties, fluopyram has a higher tendency to accumulate in roots than to be transported from roots to shoots. However, the gradual increase in individual residue levels over time can be attributed to the stability of fluopyram, which does not degrade and instead accumulates in the plant tissue. Therefore, fluopyram has the potential to accumulate through uptake and translocation.

### 3.5. BCFs and Management Concentration of Fluopyram in Soil

The 95% confidence intervals of the BCF of scallion were determined from soil residues at DAP 0 and scallions at DAP 48, as shown in Table 5. The BCFs of scallions were in the range of 0.21–0.24 and 0.14–0.18 in trials A and B, respectively, which were lower than that of bell pepper leaves (0.32) and higher than that of tomato leaves (0.12) [7]. These results indicate that the uptake and accumulation capacity of fluopyram by scallions was intermediate between that of bell pepper leaves and tomato leaves.

Based on the MRL in the crop and BCFs in this study, the management concentration in soil (MC_soil_) was determined, which is the maximum tolerable level in soil caused by pesticides used during the cultivation of the preceding crop, thereby minimizing residual pesticide levels in rotational crops grown in contaminated soils and producing safe agricultural commodities. In the Republic of Korea, the MRL for fluopyram arising from unintentional contamination is set at 0.2 mg/kg for leaf-and-stem vegetables, including scallions. The minimum MC_soil_ for fluopyram was calculated as 0.83 mg/kg from the upper 95% confidence limit of the BCF. Therefore, the management concentration for fluopyram in the soil is proposed as less than 0.8 mg/kg. This threshold for fluopyram in the soil is likely to be set as a precautionary measure to ensure that scallions grown in soil contaminated with fluopyram will not be exposed to the MRL in scallions. If the concentration of fluopyram in the soil is found to be higher than the proposed management level of 0.8 mg/kg, appropriate measures should be taken to mitigate the associated risks. These measures may include delaying planting of seedlings, tillage, and soil dressing. These actions are generally taken to reduce the level of fluopyram in the soil to an acceptable level, in order to protect the rotational crops and the environment.

## 4. Conclusions

This study investigated the residue pattern of fluopyram, in terms of the bioconcentration factor, in soil and its uptake by scallions during greenhouse cultivation. Residues in the soil and scallions were measured using a modified QuEChERS method coupled with HPLC-MS/MS and validated according to the European SANTE/12682/2019 guidelines. Fluopyram residues in soil showed limited dissipation during cultivation, with a DT_50_ of 87–231 days. The DT_50_ values were found to be higher than that reported for other crops in the previous literature, indicating that fluopyram may be a persistent pollutant in the environment. Fluopyram in soil was taken up by scallions through the roots, but residual levels in scallions during cultivation decreased due to an increase in plant weight, which caused a dilution effect. The peak BCF was estimated as 0.24. Using the BCFs, the MC_soil_ was determined as 0.8 mg/kg to avoid exceeding the MRL (0.2 mg/kg) for scallions. Continuous monitoring and management of residual fluopyram in soil is necessary to ensure compliance with MRLs, since this fungicide has the potential to persist for extended periods of time.

## Figures and Tables

**Table 1:** Physico-chemical properties of fluopyram.

Pesticide	Fluopyram
Structure	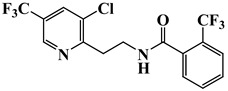
Chemical name	*N*-{2-[3-chloro-5-(trifluoromethyl)-2-pyridyl]ethyl}-α,α,α-trifluoro-o-toluamide
Molecular weight	396.72
Solubility	In water 16.0 mg/L (20–25 °C), soluble in acetone and methanol (>250 g/L)
Log K_ow_	3.3 (pH 6.5)

**Table 2:** Physico-chemical properties of soil.

pH (1:5)	EC ^1^ (dS/m)	OM ^2^ (g/kg)	Exchangeable Cations (cmol/kg)			CEC ^3^ (cmol/kg)	Particle Distribution (%)			Soil Texture
			K	Ca	Mg		Sand	Silt	Clay	
4.3	4.6	74.8	1.1	6.5	1.7	21.2	32.2	50.8	17.1	Silt loam

^1^ Electrical conductivity; ^2^ Organic matter; ^3^ Cation exchange capacity.

**Table 3:** Recovery efficiencies for fluopyram in soil and scallions.

Matrix	Fortification Level (mg/kg)	Recovery (%, Mean ± SD ^1^)	MLOQ ^2^ (mg/kg)
Soil	0.01	71.3 ± 0.4	0.01
	0.1	90.1 ± 2.2	
	1.0	91.3 ± 1.2	
Scallion	0.01	87.5 ± 0.5	0.01
	0.1	96.3 ± 1.2	

^1^ Mean ± standard deviation of triplicate samples. ^2^ Matrix-dependent limits of quantitation.

**Table 4:** Residues of fluopyram in field soil and scallions.

Days after Planting (DAP)	Residue Levels (mg/kg, Mean ± SD ^1^)			
	Soil		Scallion	
	Trial A	Trial B	Trial A	Trial B
0	0.94 ± 0.03	0.96 ± 0.04	-	-
20	-	-	0.35 ± 0.03	0.22 ± 0.01
27	-	-	0.28 ± 0.05	0.24 ± 0.02
34	0.86 ± 0.01	0.64 ± 0.02	0.27 ± 0.09	0.25 ± 0.03
41	-	-	0.27 ± 0.09	0.17 ± 0.01
48	0.80 ± 0.06	0.69 ± 0.01	0.22 ± 0.01	0.15 ± 0.01

^1^ Mean ± standard deviation of triplicate samples.

**Table 5:** Bioconcentration factors (BCFs) of fluopyram calculated from residue estimates (95% confidence interval) in soil and scallions.

Trials	C_soil_ (mg/kg)	C_plant_ (mg/kg)	BCF
A	0.90–0.99	0.21–0.22	0.21–0.24
B	0.88–1.04	0.13–0.17	0.14–0.18

## Data Availability

All related data and methods are presented in this article.
